# Additional Benefit of Chinese Medicine Formulae Including *Dioscoreae rhizome (Shanyao)* for Diabetes Mellitus: Current State of Evidence

**DOI:** 10.3389/fendo.2020.553288

**Published:** 2020-11-10

**Authors:** Lu Sun, Yuan Ming Di, Chuanjian Lu, Xinfeng Guo, Xianyu Tang, Anthony Lin Zhang, Charlie Changli Xue, Guanjie Fan

**Affiliations:** ^1^ The Second Affiliated Hospital of Guangzhou University of Chinese Medicine, Guangzhou, China; ^2^ The Second Clinical College of Guangzhou University of Chinese Medicine, Guangzhou, China; ^3^ Guangdong Provincial Hospital of Chinese Medicine, Guangzhou, China; ^4^ Guangdong Provincial Academy of Chinese Medical Sciences, Guangzhou, China; ^5^ The China–Australia International Research Centre for Chinese Medicine, School of Health and Biomedical Sciences, RMIT University, Melbourne, VIC, Australia

**Keywords:** *Dioscoreae rhizome*, *Shan yao*, Chinese medicine, diabetes, systematic review, efficacy, safety, mechanism

## Abstract

**Background:**

Chinese medicine has been used to treat diabetes symptoms for thousands of years. *Dioscoreae rhizome* or *Shanyao* is a Chinese medicinal herb that is routinely used in the treatment of diabetes mellitus (DM).

**Objective:**

The purpose of this study is to evaluate the evidence of the added benefits and safety of herbal formulae containing *Shanyao* in clinical studies and the possible mechanisms of *Shanyao* in the prevention and treatment of DM in experimental studies.

**Methods:**

We searched nine databases for randomized controlled trials (RCTs) that included *Shanyao* in the formulae in the treatment of type 2 DM. Furthermore, experimental studies on the prevention and treatment of DM by *Shanyao* in English- and Chinese-language databases were identified.

**Results:**

Fifty-three moderate quality RCTs with herbal formulae containing *Shanyao* were identified. Results from meta-analysis indicated that *Shanyao* alone or formulae containing *Shanyao* in addition to conventional treatments could benefit people with type 2 DM in lowering blood glucose, blood lipids and reducing insulin resistance. Moreover, adverse events were significantly lower in the CHM plus conventional group than those in the conventional group. *Shanyao* may exert the benefit through various mechanisms including inhibition of *α*-glucosidase and DPP-IV activity, increase of endogenous GLP-1 and immune regulating activities.

**Conclusion:**

Evidence from this review suggested that there appeared to be added clinical benefits associated with the use of *Shanyao* for DM, whether as a food supplement or as a CHM combined with hypoglycemic agents with a good safety profile.

## Introduction

Diabetes mellitus (DM) prevalence is growing at an alarming rate worldwide. According to the International Diabetes Federation, about 463 million adults worldwide suffer from DM (1). Among them, China is the country with the largest number of people suffering from DM.

Approximately 95% of the diabetic population is T2DM, followed by type 1 diabetes mellitus, gestational diabetes mellitus, and specific types of diabetes due to other causes (2). Patients with type 1 diabetes mellitus and gestational diabetes mellitus are mainly treated with insulin supplementation and replacement therapy. Specific etiology and pathogenesis of other types of DM vary, and the treatment plan is not unified. Therefore, this study focuses on patients with T2DM.

Epidemiological figures showed that the prevalence of type 2 diabetes mellitus (T2DM) in adults aged 18 and over in China is 10.9% and that of pre-diabetes is 35.7% (3). T2DM is a chronic progressive disease. Da Qing research, a registry cohort study with long term follow-up in China showed that 67.7% of pre-diabetic people will develop DM after six years without early intervention, but the risk of T2DM in the combined diet and exercise group can be reduced by 42% (4). UK Prospective Diabetes Study suggested that strict glycemic control can reduce the risk of diabetic complications (5). Early active management can be more beneficial for diabetic people, which coincides with the idea of “cure disease before disease onset” in traditional Chinese medicine (TCM).

Treating diabetes by conventional medicine includes oral and injectable hypoglycemic agents. Diabetic patients often need a combination of several drugs. Common concerns of doctors and diabetic patients include hypoglycemia, gastrointestinal discomfort, inconvenience of missing medicines and injectable medicines. The Chinese Diabetes Society (CDS) guidelines of 2017 presented information of TCM for diabetes and recommended the use of TCM (6). In China, it has been observed that doctors in the field of diabetes are also looking for diverse treatments including TCM (6).

In TCM, DM belongs to the category of disease called “*xiao ke* 消渴”. Disease characteristics include thirst, excessive drinking, polyuria, and weight loss. The pathogenesis of DM in TCM is *qi* and *yin* deficiency and excessive dryness and heat. Commonly used formulae by TCM practitioners include *Liu wei di huang wan* 六味地黄丸, *Shen qi wan*肾气丸, and *Ba wei wan*八味丸 (7). *Shanyao* (*Dioscoreae rhizome*) is the root of *Dioscorea opposita* Thunb. (8); it is one of the main herbs in the above formulae. *Shanyao* has been widely used in the treatment of *xiao ke*消渴or diabetes since the ancient times. It is a popular herb and can also be consumed as a type of food. Active ingredients of *Shanyao* include polysaccharides, flavonoids, allantoin, choline, and dioscin (9–14).

This study will review evidence of *Shanyao* for DM from clinical research and experiment research results from Chinese- and English-language databases and present the evidence on added benefits and safety of herbal formula containing *Shanyao* in clinical studies. Possible mechanisms of *Shanyao* in the prevention and treatment of DM in experimental studies are also investigated.

## Methods

### Systematic Review of Clinical Trials

#### Search Strategy

We searched English- and Chinese-language databases and followed the methods outlined in the Cochrane Handbook of Systematic Reviews (15). English-language databases included PubMed, ExcerptaMedica Database (Embase), Cumulative Index of Nursing and Allied Health Literature (CINAHL), Cochrane Central Register of Controlled Trials (CENTRAL), including the Cochrane Library, and Allied and Complementary Medicine Database (AMED); Chinese-language databases included China SinoMed, China National Knowledge Infrastructure (CNKI), Chongqing VIP (CQVIP), and Wanfang. Databases were searched from inception to March 2019. No restrictions were applied.

Search terms were grouped into three blocks: 1) intervention (formulae including *Shanyao*, *dioscoreae rhizome*); 2) clinical condition (including type 2 diabetes mellitus); and 3) trial design (including clinical trial, randomized controlled trial).

We also searched reference lists of previous systematic reviews and included studies. Clinical trial registries were also searched including the Australian New Zealand Clinical Trial Registry (ANZCTR), Chinese Clinical Trial Registry (ChiCTR), European Union Clinical Trials Register (EU-CTR) and USA National Institutes of Health register (ClinicalTrials.gov). When required, we contacted trial investigators by email or telephone to obtain data. If we didn’t receive a response after four weeks, we marked the unknown information ‘not available’.

Protocol of the review was registered with the PROSPERO international prospective register of systematic reviews (CRD 42019145668).

#### Study Inclusion Criteria

##### Study design

Randomized controlled trials (RCTs) were eligible.

##### Participants

Adults were diagnosed with T2DM using the following guidelines: 1999 World Health Organization (WHO) (16), Chinese Diabetes Society (17, 18), American Diabetes Association (2), or a description of diagnostic criteria including:

- Fasting blood glucose (FBG, defined as no caloric intake for at least 8 h) ≥126 mg/dl (7.0 mmol/L),
- Or 2-h plasma glucose (PG) ≥200 mg/dl (11.1 mmol/L) during an oral glucose tolerance test (OGTT). The test should be performed as described by the WHO, using a glucose load containing the equivalent of 75 g anhydrous glucose dissolved in water.
- Or A1C ≥6.5% (48 mmol/mol). The test should be performed in a laboratory using a method that is National Glycohemoglobin Standardization Program certified and standardized to the Diabetes Control and Complications Trial assay.
- Or in a patient with classic symptoms of hyperglycemia or hyperglycemic crisis, a random plasma glucose ≥200 mg/dl (11.1 mmol/L). In the absence of unequivocal hyperglycemia, results should be confirmed by repeat testing.

##### Type of Interventions

CHM, other CM therapies (for example, Chinese medicine dietary therapy), and integrative medicine such as CHM plus hypoglycemic drugs; all the interventions should include *Shanyao*.

##### Type of Controls

Conventional therapies recommended in guidelines, including pharmacotherapy, diet therapy and lifestyle interventions.

##### Outcomes

The primary outcome measures were:

- Blood glucose tests (fasting blood glucose, post-prandial blood glucose, hemoglobin a_1c_);
- Adverse events (AEs);

The secondary outcomes were:

- Blood lipid metabolism indicators (triglyceride, cholesterol, low-density lipoprotein, high-density lipoprotein);
- *β*-cell function indicators: fasting serum insulin (Fins) the unit of FINS is pmol/L or μu/ml will be included (pmol/L = μu/ml × 6.965); HOMA-IR(IR) = FBG × FINS/22.5; HOMA-IS (IS) = 1/(Fins × FPG);
- Body Mass Index (BMI).

#### Study Exclusion Criteria

- Quasi-randomized controlled trials;
- Prediabetic state;
- Type 1 diabetes;
- Gestational diabetes;
- Other specific types of diabetes included in the American Diabetes Association and Chinese Diabetes Society:
  - Genetic defects of beta-cells
  - Genetic defects in insulin action
  - Diseases of the exocrine pancreas
  - Endocrinopathies
  - Drug or chemical induced diabetes
  - Infections
  - Uncommon forms of immune-mediated diabetes
  - Other genetic syndromes sometimes associated with diabetes
- Diabetic complications and comorbidities;
- Integrative medicine studies that used different therapies in the intervention group and control group;
- If the control group uses a form of Chinese medicine.

#### Data Extraction and Management

Search results were synthesized by removing duplicates, followed by screening of titles and abstracts by LS and YD. Full texts were obtained and screened by two reviewers (LS and YD). Eligible studies satisfying the inclusion criteria were extracted using EpiData software (EpiData Association, Odense, Denmark). LS and YD extracted the data from the included studies independently and double-checked the data to obtain information on authors, publication year, title, journal, participants’ characteristics, sample size, methodological details, intervention details, treatment duration, outcome measures, and AEs.

#### Assessment of Risk of Bias in Included Studies

Risk of bias was assessed using the Cochrane Collaboration’s procedures (15). RevMan software (Version 5.2.4, Copenhagen: The Nordic Cochrane Centre, The Cochrane Collaboration, 2012) was used for risk of bias analysis. Items of bias assessed included sequence generation, allocation concealment, blinding of participants, blinding of personnel, blinding of outcome assessment, incomplete outcome data, selective outcome reporting and other bias including baseline imbalance and funding. Egger’s test was used to assess publication bias. Publication bias was assessed when the subgroup included more than 10 studies.

Risk of bias assessment was conducted by two independent reviewers (LS and YD) and disagreement was resolved by discussion or consultation with a third person (TZ).

#### Data Analysis

Continuous outcomes were presented as mean difference (MD) with 95% confidence interval (CI) between two groups, whereas dichotomous data were presented as relative risk (RR) with 95% CI. Stata software (13.0) was used for data analysis. Considering heterogeneities among trials, all meta-analyses were performed with random effects model. Heterogeneities between studies was estimated by *I* ^2^. An *I^2^* score greater than 50% was considered to indicate substantial heterogeneity.

Predefined comparisons in the meta-analysis were as follows: (1) CHM plus hypoglycemic agents *versus* hypoglycemic agents, (2) CHM plus lifestyle intervention *versus* lifestyle intervention, (3) CHM diet therapy plus lifestyle intervention *versus* lifestyle intervention.

Subgroup analysis were performed where possible, including studies with low risk for sequence generation, FBG level at baseline(6–≤8 mmol/L, 8–10 mmol/L, ≥10 mmol/L), patient age groups (18–40 years, 41–64 years, >65 years), BMI (normal < 24 kg/m^2^, overweight ≥ 24–28 kg/m^2^, obese ≥ 28 kg/m^2^), disease duration (<5 years, ≥5–10years, ≥10 years), treatment duration (≤3 months, 3–6 months, and ≥6 months), comparator drugs class and CM syndrome differentiation (19–23).

The Grading of Recommendations Assessment, Development and Evaluation (GRADE) approach was used to assess the quality of evidence.

### Pharmacological Research Evidence of *Shanyao* for DM

The constituent compounds were identified by searching herbal monographs, high quality reviews of CHM, pharmacopoeia of the People’s Republic of China (24), and PubMed. To identify preclinical publications a literature search of PubMed and China National Knowledge Infrastructure was undertaken. The search strategy included the terms for *Shanyao* and its constituent compounds and T2DM. Relevant data were extracted, and a summary of the findings are reported here.

## Results

### Modern Literature Results

#### Description of Included Studies

##### Search Results

**Figure 1:**
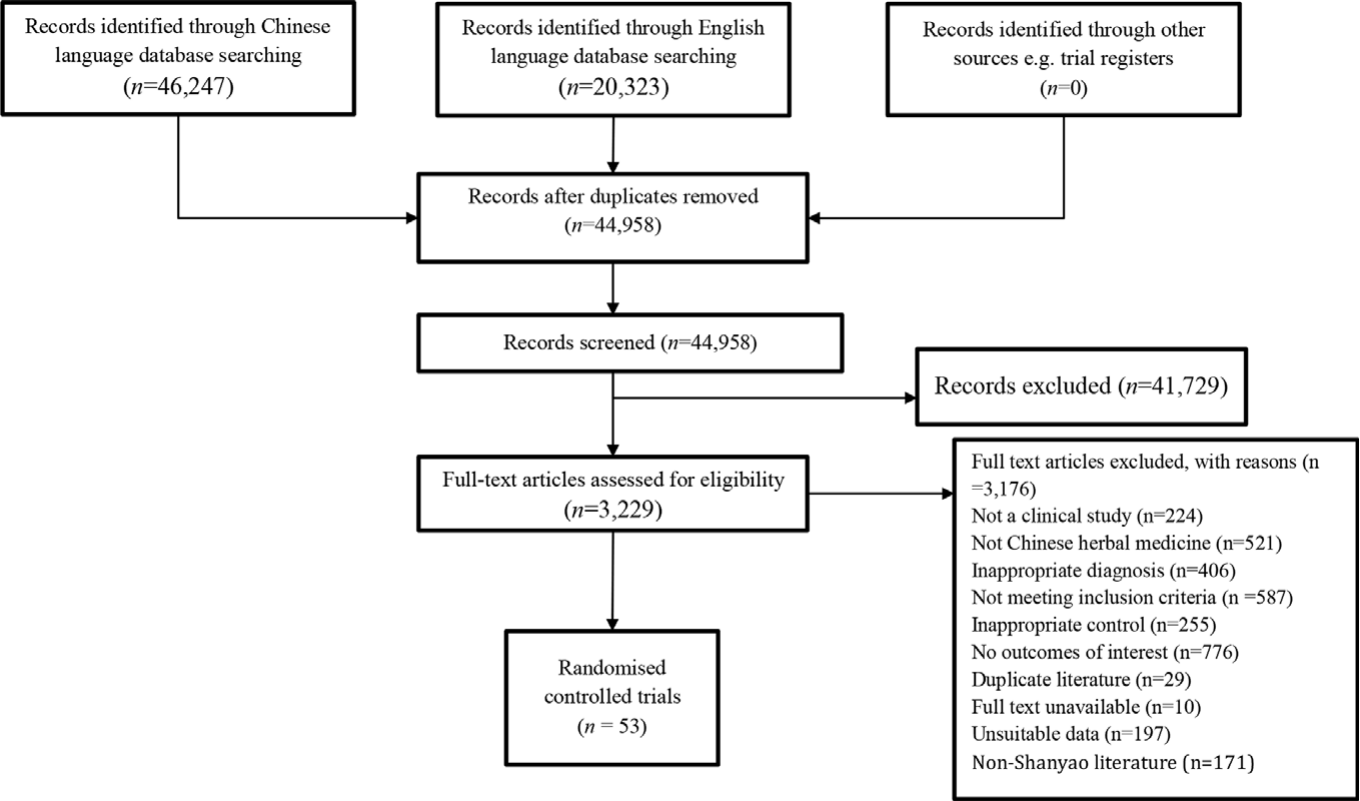
Flow chart of study selection process.

Our search identified 44,958 articles in the included databases. Fifty-three (53) RCTs involving 4,905 participants were included in the systematic review (25–77). The screening process is shown in **Figure 1**.

##### Characteristics of the Included Studies

All studies were randomized, parallel-group, controlled trials conducted in China between 2002 and 2018. One study published was in English (44) and the rest in Chinese language. All studies included participants diagnosed in accordance with the 1999 WHO, Chinese Diabetes Society or American Diabetes Association diagnostic criteria for T2DM. In total, 4,905 participants were included in these RCTs; participants’ age ranged from 45 to 74 years. Duration of T2DM ranged from 1 week to 20 years. Treatment duration ranged from 2 to 24 weeks. Only one study had a follow-up for 30 weeks (65). Characteristics of included studies are summarized in **Table 1**.

**Table 1:** Basic characteristics of the included studies in modern literature.

NO.	Study	Sample size	Sample size	Mean age(y)	Mean age(y)	Treatment	Control	Duration(w)	FBG	2hPG	HbA_1c_	TG	TC	LDL	HDL	BMI	FINS	IR	IS	AEs
		(I)	(C)	(I)	(C)															
1	Bai (42)	40	30	48.27	49.23	SMF&Bigu&Sulf	Bigu/Sulf	4	√	√	×	√	√	×	×	×	×	×	×	×
2	Cao et al. (77)	68	68	48	47	SMF&Bigu	Bigu	8	√	√	√	√	√	×	×	×	×	×	×	√
3	Cao and Zou (76)	54	54	56.7	54.5	Baihurenshen Formula&Sulf	Sulf	8	√	√	×	×	×	×	×	×	√	√	√	√
4	Chen (30)	24	26	60.33	61.35	a-Glucosidase or Insulins	a-Glucosidase/Insulins	6	√	√	√	√	√	√	√	×	×	×	×	√
5	Cui and Lou (75)	40	40	56.75	52.57	SMF&Bigu&Sulf	Bigu&Sulf	8	√	√	√	×	×	×	×	×	×	×	×	×
6	Fan et al. (74)	40	40	42–74	40–73	Shenqijiangtang Granule&Bigu	Bigu	12	√	√	√	×	×	×	×	×	×	×	×	×
7	Gao et al. (73)	90	90	58.9	57.6	Shenqijiangtang Capsule&Bigu	Bigu	12	√	√	√	√	√	√	√	×	×	×	×	×
8	Hou (72)	30	30	46.12	46.78	seld-made Formula&Bigu&a-Glucosidase	Bigu&a-Glucosidase	8	√	√	√	√	√	√	√	√	√	√	×	√
9	Li (25)	45	45	69.76	69.44	Chinese medicine diet therapy	No treatment	12	√	√	×	√	√	√	√	×	×	×	×	×
10	Li (32)	30	30	NS	NS	SMF&Bigu&a-Glucosidase	Bigu&a-Glucosidase	4	√	√	√	×	×	×	×	×	×	×	×	√
11	Li et al. (71)	102	100	57.20	58.46	Jiangtang Capsule&Sulf	Sulf	4	√	√	×	√	√	√	√	×	×	×	×	×
12	Lin (40)	20	20	56.2	54.8	Zhibodihuang Formula&Bigu	Bigu	4	√	√	√	×	×	×	×	×	×	×	×	×
13	Liu et al. (70)	29	29	46.5	46.9	SMF&Bigu	Bigu	12	√	×	√	√	√	√	√	×	×	×	×	×
14	Liu et al. (69)	30	30	NS	NS	SMF&DPP4	DPP4	12	√	√	×	×	×	×	×	×	√	×	×	×
15	Liu (29)	30	30	57.3	56.7	SMF&Bigu&Sulf	Bigu&Sulf	12	√	√	√	√	√	√	√	×	×	×	×	√
16	Lou and Zhao (68)	52	50	53.5	54	SMF&Bigu	Bigu	8	√	√	√	×	×	×	×	×	×	×	×	×
17	Lv et al (35)	60	60	54.61	55.77	Yuye Formula&insulin	insulin	4	√	√	×	√	√	√	√	×	×	×	×	×
18	Lv et al. (67)	45	30	58.9	58.8	SMF&a-Glucosidase	a-Glucosidase	4	√	√	√	×	×	×	×	×	×	×	×	×
19	Peng et al. (66)	25	25	57	58	SMF&Bigu&a-Glucosidase	Bigu&a-Glucosidase	8	√	√	×	√	√	√	√	×	×	×	×	×
20	Peng and Xu (36)	68	68	74.86	75.01	Shenqi JiangTang Pill&a-Glucosidase	a-Glucosidase	7	√	√	×	×	×	×	×	×	×	×	×	×
21	Shang et al. (65)	30	30	48.36	47.97	SMF&CSII	CSII	8	×	×	×	×	×	×	×	×	√	√	×	×
22	Tang and Li (64)	69	69	51.6	50.4	Liuweidihuang Pill&Bigu	Bigu	12	√	√	√	√	√	√	√	×	√	√	×	√
23	Wang and Wang (27)	29	27	59.1	59.2	SMF&Bigu&Sulf	Bigu&Sulf	8	√	√	√	√	√	√	√	×	×	×	√	×
24	Wang (37)	30	30	68.2	67.3	SMF&CSII	CSII	2	√	×	√	√	√	√	√	×	×	×	×	√
25	Wang (43)	30	30	50.3	53.2	Gankujiangtang Formula&Bigu	Bigu	12	√	√	√	√	√	√	√	×	√	√	×	√
26	Wang (33)	30	30	50	53.7	Zhibodihuang Formula&Sulf	Sulf	4	√	√	×	×	×	×	×	×	√	√	√	×
27	Wu (28)	20	18	51.85	54.78	SMF&Bigu	Bigu	8	√	√	√	√	√	×	×	√	×	×	√	√
28	Xie and Lu (63)	135	133	35-64	36–60	Dihuangjiangtang pill&Bigu	Bigu	12	×	×	×	√	√	√	√	×	×	×	×	×
29	Xu et al. (62)	20	20	54	55	Shenlinbaizhu Formula&insulin	insulin	12	×	×	×	×	×	×	×	√	×	×	×	×
30	Yang (39)	30	30	NS	NS	Qianwenwu Formula&Bigu	Bigu	9	√	√	√	√	√	√	√	×	×	×	×	×
31	Yu et al. (60)	40	40	56.2	55.4	Taipingtangke Pill&Bigu	Bigu	12	√	√	√	×	×	×	×	×	×	×	×	×
32	Yu et al. (61)	30	30	32-64	34–67	SMF&Bigu	Bigu	8	√	√	×	×	×	×	×	×	×	×	×	×
33	Zhang et al. (59)	30	30	46.4	47.3	SMF&Bigu	Bigu	4	√	√	√	×	×	×	×	×	×	×	×	×
34	Zhang et al. (52)	32	30	54.4	56.5	SMF&Bigu	Bigu	8	√	√	√	×	×	×	×	×	×	×	×	√
35	Zhang et al. (57)	36	20	42–59	44–57	SMF&Bigu	Bigu	4	√	√	√	×	×	×	×	×	×	×	×	×
36	Zhang et al. (58)	100	50	52.6	50.5	SMF&Bigu&Sulf	Bigu&Sulf	8	√	√	×	√	√	√	√	×	√	×	×	√
37	Zhang and Pan (56)	120	60	46.5	46.4	Shendishengjin Capsule&Sulf	Sulf	8	√	√	×	√	√	√	√	×	×	×	×	√
38	Zhang (41)	30	30	NS	NS	SMF&Bigu&a-Glucosidase	Bigu&a-Glucosidase	8	√	√	√	×	×	×	×	×	×	×	×	√
39	Zhang (38)	32	31	54	56	SMF&Bigu&a-Glucosidase	Bigu&a-Glucosidase	12	√	√	√	×	×	×	×	×	×	×	×	×
40	Zhang (26)	34	32	51	49	self-made Formula	No treatment	4	√	√	×	×	×	×	×	×	×	×	×	√
41	Zhang (34)	48	44	52.3	54.2	Jiaweishenqidihuang Formula&Bigu	Bigu	12	√	×	√	√	√	√	√	√	√	√	×	×
42	Zhang et al. (55)	30	30	45.6	46.9	SMF&Sulf	Sulf	8	√	√	√	×	×	×	×	×	√	×	×	√
43	Zhang et al. (54)	93	79	NS	NS	Xiaotangping Capsule&Bigu	Bigu	12	√	√	√	√	√	√	√	×	×	×	√	×
44	Zhang (31)	20	20	56.9	57.1	SMF&insulin	insulin	12	√	√	√	√	√	√	√	×	×	×	×	×
45	Zhang et al. (44)	109	110	56.9	57.1	Shen-Qi formula & insulin	insulin	12	√	√	√	√	√	√	√	×	×	×	×	√
46	Zhang et al. (53)	48	50	55.2	53.1	SMF&insulin	insulin	ns	√	×	√	×	×	×	×	×	×	×	×	√
47	Zhao and Song (51)	70	68	56.5	53.5	SMF&Sulf&Bigu	Sulf/Bigu	24	√	√	√	×	×	×	×	×	√	×	×	√
48	Zhou et al. (50)	50	50	40–74	41–72	SMF&Sulf	Sulf	12	√	√	×	×	×	×	×	×	×	×	×	×
49	Zhou and Dong (49)	30	30	59.2	58.6	Yiqiyangyin Capsule&Sulf	Sulf	4	√	√	√	×	×	×	×	×	×	×	×	×
50	Zhou and Shen (48)	20	20	60	59.8	SMF&Routine hypoglycemic agents	Routine hypoglycemic agents	12	√	√	√	×	×	×	×	×	×	×	×	×
51	Zhou et al. (47)	124	124	NS	NS	Erban Formula&Bigu	Bigu	12	√	√	√	×	×	×	×	×	×	×	×	×
52	Zhu and Li (45)	30	30	74.2	76.8	Jiangtangqing Granule&insulin secretagogues&a-Glucosidase	insulin secretagogues&a-Glucosidase	12	√	×	√	√	√	√	√	×	√	×	×	√
53	Zhu et al. (46)	35	35	51.7	53	SMF&Bigu&Sulf	Bigu&Sulf	12	√	√	×	×	×	×	×	×	×	×	×	√

C, control; I, intervention; CHM, Chinese herbal medicine; F, female; M, male; w, week/weeks; y, year/years; Sulf, Sulfonylureas; Bigu, Biguanides; CSII, continuous subcutaneous insulin infusion; TZDs, Thiazolidinediones; DPP-4, Dipeptidyl Peptidse-4; SMF, self-made Formula.

Fifty-three (53) RCTs assessing CHM as food or integrative medicine for T2DM were identified from the search. 51 studies assessed the combination of CHM with conventional medication (integrative medicine) (27–77). One study compared CHM to lifestyle intervention (26); one study compared Chinese medicine diet therapy to lifestyle intervention (25).

Ninety-eight herbs were used in the formulae, and the most commonly used herb used with *Shanyao* 山药 were*huangqi* 黄芪 (42 studies), *shengdihuang* 生地黄 (30 studies), *gegen* 葛根 (28 studies)*, tianhuafen* 天花粉 (28 studies), *danshen* 丹参 (26 studies)*, fuling* 茯苓 (23 studies), *maimendong* 麦门冬 (22 studies)*, shanzhuyu* 山茱萸 (21 studies), and *huanglian* 黄连 (16 studies).

Comparators included pharmacologic therapy and lifestyle intervention. Pharmacologic therapy used in the included RCTs includes biguanides, sulfonylureas, thiazolidinediones, a-glucosidase inhibitors, DPP-4 inhibitors, and insulins. Lifestyle management of T2DM includes diabetes self-management education and support, medical nutrition therapy, physical activity, and psychosocial care.

CM syndrome differentiation was described in thirty-four studies (26, 28, 29, 31–41, 43, 46, 48–50, 52, 53, 55, 57, 59–62, 64, 67, 69, 71, 74, 75, 77). The most common syndromes described in the studies include *qi* and *yin* deficiency 气阴两虚, *yin* deficiency and excessive heat 阴虚热盛, damp heat retention 湿热阻滞, spleen deficiency 脾虚, phlegm-dampness, and blood stasis 痰湿血瘀.

#### Risk of Bias in the Included Studies

**Figure 2:**
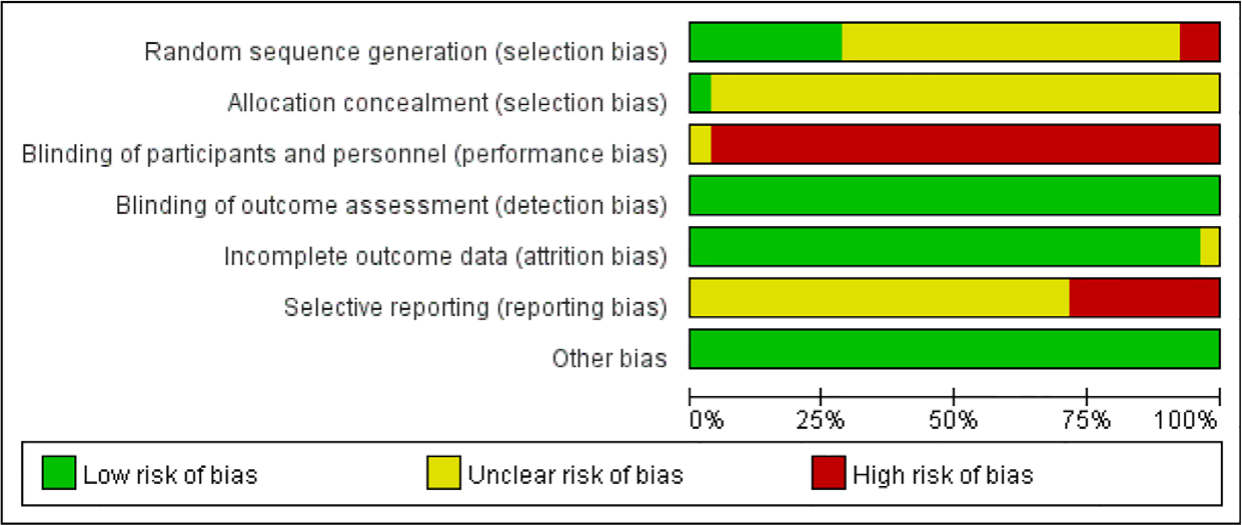
Risk of bias of included studies.

**Figure 3:**
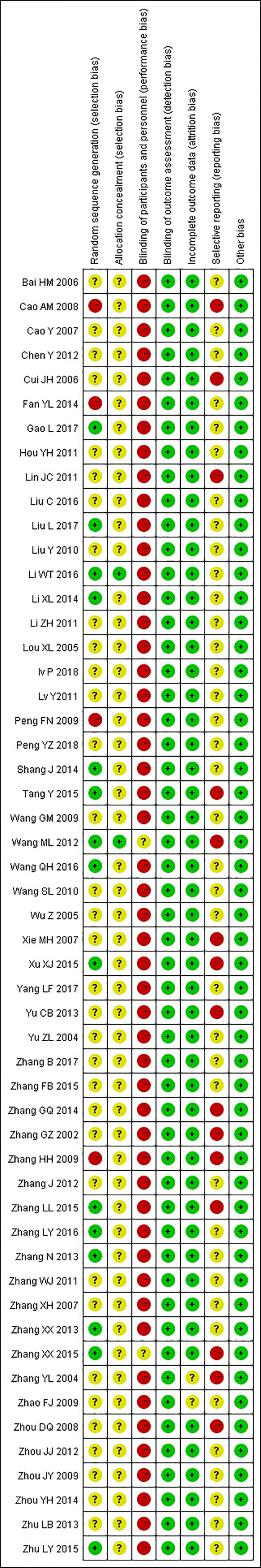
Risk of bias summy of included studies.

The results of the risk of bias judgements are presented in **Figure 2** and **Figure 3**. All included 53 studies were randomized; 15 trials described the process of random sequence generation (25, 26, 31, 32, 34, 37, 38, 43, 44, 46, 62, 64, 65, 69, 73); two trials implemented allocation concealment (25, 37). One study was described as “single-blind“ trial with no further details (37). Considering all included outcome measures were objective outcomes, blinding of assessors has low risk of influencing the outcome measures and was judged as low risk of bias in all included studies. Twelve trials did not report on all outcome measures described in the *Methods* section (37, 40, 44, 53, 56–58, 60, 62, 63, 75, 77), three trials reported on AEs which were not included in the methods section (38, 50, 64). Publication bias was not detected (Egger’s test, t = −0.71, *P* = 0.48). The overall methodological quality of the included studies was moderate.

#### Effects of Intervention

##### CHM Plus Hypoglycaemic Agents Versus Hypoglycaemic Agents

###### Fasting Blood Glucose

Forty-eight RCTs including 4,375 participants assessed the effects of CHM plus hypoglycemic agents *versus* hypoglycemic agents alone; these studies used the same hypoglycemic agents in both groups (27–61, 64, 66–77). Treatment duration ranged from 2 to 24 weeks. Different classes of hypoglycemic agents were used across studies including biguanides, sulfonylureas, *α*-Glucosidase inhibitors, DPP-4 inhibitors and insulins. Specific hypoglycemic agents include metformin, gliclazide, glibenclamide, gliquidone, glimepiride, acarbose, sitagliptin, and insulin.

The integrative use of CHM plus hypoglycemic agents was superior to hypoglycemic agents alone at reducing FBG levels at the end of treatment [MD −0.93 (−1.10, −0.76); I^2^ = 84.2%], although heterogeneity was high. Meta-analysis of studies assessed as low risk of bias for sequence generation produced a similar result to the overall results with reduced heterogeneity [11 RCTs, 1,050 participants, MD −0.68 (−0.89, −0.47); I^2^ = 67.9%] (31, 32, 34, 37, 38, 43, 44, 46, 64, 69, 73).

Subgroup analyses based on comparator drug class, patient age groups, baseline levels of FBG, CM syndrome differentiation, treatment duration, disease duration, and baseline levels of BMI all showed significant differences between groups (**Table 2**).

**Table 2:** Summary of meta-analysis results and sub-group analysis.

Treatment *Vs*. Comparison	Outcomes(unit)	Group	Subgroup	No. of Studies	MD[95% CI]	I^2^
**CHM plus Hypoglycaemic agents *versus* Hypoglycaemic agents**	FBG (mmol/L)	All studies	All studies	48(4,375)	−0.93[−1.10, −0.76]*	84.2%
		Risk of bias SG	Low risk of bias SG	11(1,050)	−0.68[−0.89, −0.47]*	67.9%
		comparator Drug class	Biguanides	18(1,722)	−0.91 [−1.11, −0.70]*	63.8%
			Sulfonylureas	7(770)	−0.92 [−1.58, −0.26]*	91.9%
			Insulin	4(477)	−0.96[−1.57, −0.35]*	85.0%
			Biguanides + Sulfonylureas	5(416)	−1.18[−1.46, −0.89]*	0.0%
			Biguanides+ a-Glucosidase	5(293)	−0.97[−1.69, −0.24]*	96.4%
		Age of patients	41–64 years	36(3,219)	−0.79[−1.02, −0.57]*	86.7%
			>65 years	4(504)	−1.28[−1.51, −1.06]*	0.0%
		FBG level at baseline	8–10 mmol/L	27(2,413)	−0.77[−0.95, −0.59]*	74.9%
			≥10 mmol/L	21(1,962)	−1.15[−1.48, −0.82]*	89.2%
		CM syndrome differentiation	*qi* and *yin* deficiency	23(1,886)	−0.97[−1.23, −0.72]*	81.6%
			*yin* deficiency and excessive heat	6(430)	−0.82[−1.05, −0.59]*	23.0%
			Spleen deficiency	3(158)	−0.57[−1.10, −0.04]*	87.6%
		treatment duration	≤3 months	46(4,139)	−0.91[−1.08, −0.73]*	83.9%
			≥6 months	1(138)	−1.00[−1.34, −0.66]*	NA
		Disease duration	<	14(1,093)	−0.80[−1.09, −0.51]*	77.2%
			≥5–10 years	10(924)	−1.07[−1.55, −0.59]*	90.7%
			≥10 years	10(1,001)	−0.88[−1.14, −0.62]*	72.5%
		BMI level at baseline	≥24–28 kg/m^2^	3(193)	−0.53[−0.87, −0.19]*	0.0%
	2hPG (mmol/L)	All studies	All studies	43(4,004)	−1.46[−1.73, −1.20]*	86.2%
		Risk of bias SG	Low risk of bias SG	9(895)	−1.64[−2.27, −1.02]*	91.8%
		comparator Drug class	Biguanides	16(1,572)	−1.36[−1.62, −1.11]*	44.3%
			Sulfonylureas	7(770)	−1.60[−2.48, −0.72]*	89.6%
			Insulin	3(379)	−1.71[−3.37, −0.05]*	91.2%
			Biguanides + Sulfonylureas	5(416)	−1.12[−2.22, −0.02]*	86.4%
			Biguanides + a-Glucosidase	5(298)	−1.58[−2.41, −0.74]*	94.7%
		Age of patients	41–64 years	33(2,968)	−1.45[−1.78, −1.12]*	86.7%
			>65 years	2(384)	−1.65[−2.92, −0.39]*	94.1%
		FBG level at baseline	8–10 mmol/L	24(2 200)	−1.38[−1.74, −1.03]*	85.6%
			≥10 mmol/L	19(1,804)	−1.56[−1.98, −1.15]*	87.3%
		CM syndrome differentiation	*qi* and *yin* deficiency	20(1,633)	−1.25[−1.67, −0.84]*	85.4%
			yin deficiency and excessive heat	6(430)	−1.51[−2.16, −0.87]*	71.4%
			Spleen deficiency	3(158)	−0.96[−1.29, −0.63]*	44.3%
		treatment duration	≤3 months	42(3,866)	−1.46[−1.73, −1.18]*	86.3%
			≥6 months	1(138)	−1.73[−2.07, −1.39]*	NA
		Disease duration	<5 years	14(1,093)	−1.57[−2.00, −1.15]*	75.9%
			≥5–10 years	9(866)	−1.35[−2.11, −0.59]*	92.7%
			≥10 years	7(783)	−1.38[−2.16, −0.61]*	92.4%
		BMI level at baseline	≥24–28 kg/m^2^	2(98)	−1.35[−2.25, −0.46]*	46.6%
	HbA_1c_ (%)	All studies	All studies	35(3,009)	−0.84[−1.05, −0.64]*	88.2%
		Risk of bias SG	Low risk of bias SG	9(920)	−0.74[−1.16, −0.32]*	93.9%
		comparator Drug class	Biguanides	17(1,665)	−0.85[−1.04, −0.66]*	75.1%
			Sulfonylureas	2(120)	−.65[−1.07, −0.24]*	0.0%
			Insulin	3(357)	−1.43[−2.69, −0.18]*	94.8%
			Biguanides + Sulfonylureas	3(196)	−0.35[−0.68, −0.02]*	0.0%
			Biguanides + a-Glucosidase	4(248)	−0.85[−1.68, −0.02]*	96.7%
		Age of patients	41–64 years	27(2,209)	−0.77[−0.99, −0.55]*	85.1%
			>65 years	3(368)	−0.95[−1.76, −0.14]*	77.2%
		FBG level at baseline	8–10 mmol/L	22(1,935)	−0.73[−0.97, −0.50]*	87.5%
			≥10 mmol/L	13(1,074)	−1.04[−1.41, −0.67]*	86.9%
		CM syndrome differentiation	*qi* and *yin* deficiency	17(1,208)	−0.67[−0.91, −0.44]*	72.8%
			*yin* deficiency and excessive heat	4(300)	−0.82[−1.21, −0.43]*	69.3%
			Spleen deficiency	3(158)	−1.07[−2.21, 0.07]	97.3%
		treatment duration	≤3 months	33(2,773)	−0.82[−1.04, −0.61]*	88.5%
			≥6 months	1(138)	−0.60[−0.99, −0.21]*	NA
		Disease duration	<5 years	11(717)	−0.72[−0.97, −0.47]*	64.6%
			≥5–10 years	6(532)	−0.91[−1.34, −0.48]*	79.8%
			≥10 years	9(893)	−0.90[−1.39, −0.42]*	94.0%
		BMI level at baseline	≥24–28 kg/m^2^	3(193)	−0.30[−0.62, 0.02]	39.9%
	TG (mmol/L)	All studies	All studies	24(2,582)	−0.40[−0.51, −0.29]*	79.2%
		Risk of bias SG	Low risk of bias SG	7(792)	−0.36[−0.50, −0.22]*	50.5%
		comparator Drug class	Biguanides	10(1,205)	−0.39[−0.53, −0.24]*	65.5%
			Sulfonylureas	2(382)	−0.06[−0.12, 0.00]	0.0%
			Insulin	3(379)	−0.46[−0.74, −0.18]*	56.5%
			Biguanides + Sulfonylureas	3(266)	−0.37[−0.58, −0.17]*	40.0%
			Biguanides + a-Glucosidase	2(110)	−0.37[−0.52, −0.21]*	0.0%
		Age of patients	41–64 years	20(2,230)	−0.37[−0.48, −0.26]*	73.3%
			>65 years	2(120)	−0.59[−0.81, −0.37]*	0.0%
		FBG level at baseline	8–10 mmol/L	13(1,342)	−0.36[−0.45, −0.27]*	32.1%
			≥10 mmol/L	10(972)	−0.43[−0.64, −0.22]*	88.1%
		CM syndrome differentiation	*qi* and *yin* deficiency	8(773)	−0.34[−0.52, −0.15]*	76.1%
			*yin* deficiency and excessive heat	2(198)	−0.19[−0.33, −0.05]*	0.0%
			Spleen deficiency	1(38)	−0.85[−1.73, 0.03]	NA
		Disease duration	<5 years	7(800)	−0.31[−0.45, −0.18]*	48.3%
			≥5–10years	6(612)	−0.49[−0.81, −0.17]*	92.7%
			≥10 years	6(695)	−0.49[−0.62, −0.37]*	6.8%
		BMI level at baseline	≥24–28 kg/m^2^	3(193)	−0.57[−0.87, −0.28]*	44.1%
	TC (mmol/L)	All studies	All studies	24(2,582)	−0.40[−0.58, −0.22]*	95.2%
		Risk of bias SG	Low risk of bias SG	7(792)	−0.34[−0.86, 0.19]	98.3%
		comparator Drug class	Biguanides	10(1,205)	−0.36[−0.70, −0.02]*	97.6%
			Sulfonylureas	2(382)	−0.28[−0.82, 0.25]	89.1%
			Insulin	3(379)	−0.13[−0.60, 0.34]	77.1%
			Biguanides + Sulfonylureas	3(266)	−0.69[−1.59, 0.21]	73.9%
			Biguanides + a-Glucosidase	2(110)	−0.44[−0.74, −0.15]*	0.0%
		Age of patients	41–64 years	20(2,230)	−0.39[−0.59, −0.18]*	96.0%
			>65 years	2(120)	−0.46[−0.99, 0.08]	57.8%
		FBG level at baseline	8–10 mmol/L	13(1,342)	−0.39[−0.70, −0.08]*	97.0%
			≥10 mmol/L	10(972)	−0.50[−0.66, −0.34] *	66.5%
		CM syndrome differentiation	*qi* and *yin* deficiency	8(773)	−0.40 [−0.67, −0.12] *	95.9%
			*yin* deficiency and excessive heat	2(198)	−0.77[−1.92, 0.38]	98.7%
			Spleen deficiency	1(38)	0.12[−0.38, 0.62]	NA
		Disease duration	<5 years	7(800)	−0.39[−0.94, 0.16]	96.0%
			≥5–10 years	6(612)	−0.63[−0.77, −0.50]*	76.8%
			≥10 years	6(695)	−0.16[−0.43, 0.11]	71.3%
		BMI level at baseline	≥24–28 kg/m^2^	3(193)	−0.02[−0.15, 0.10]	10.6%
	LDL (mmol/L)	All	All studies	21(2,338)	−0.45[−0.65, −0.27]*	97.4%
		Risk of bias SG	Low risk of bias SG	7(792)	−0.68[−1.10, −0.26]*	97.9%
		comparator Drug class	Biguanides	8(1,031)	−0.51[−0.88, −0.14]*	98.5%
			Sulfonylureas	2(382)	−0.06[−0.16,0.04]	0.0%
			Insulin	3(379)	−0.57[−1.18, 0.05]	97.6%
			Biguanides + Sulfonylureas	3(266)	−0.45[−0.60, −0.30]*	0.0%
			Biguanides + a-Glucosidase	2(110)	−0.39[−0.66, −0.12]*	78.1%
		Age of patients	41–64 years	17(1,986)	−0.45[−0.69, −0.21]*	97.7%
			>65 years	2(120)	−0.73[−1.82, 0.35]	78.9%
		FBG level at baseline	8–10 mmol/L	12(1,206)	−0.53[−0.83, −0.22]*	97.7%
			≥10 mmol/L	8(864)	−0.23[−0.33, −0.13]*	68.4%
		CM syndrome differentiation	*qi* and *yin* deficiency	7(637)	−0.29[−0.44, −0.15]*	75.1%
			*yin* deficiency and excessive heat	2(198)	−0.79[−2.03, 0.46]	99.4%
		Disease duration	<5 years	6(762)	−0.59[−1.08, −0.09]*	98.8%
			≥5–10 years	5(542)	−0.31[−0.46, −0.16]*	80.7%
			≥10 years	5(559)	−0.64[−1.11, −0.17]*	95.5%
		BMI level at baseline	≥24–28 kg/m^2^	2(155)	−0.27[−0.35, −0.20]*	0.0%
	HDL (mmol/L)	All	All studies	21(2,338)	0.15[0.08, 0.22]*	93.9%
		Risk of bias SG	Low risk of bias SG	7(792)	0.18[0.07, 0.29]*	93.3%
		comparator Drug class	Biguanides	8(1,031)	0.24[0.14, 0.33]*	89.6%
			Sulfonylureas	2(382)	0.18[−0.21, 0.58]	96.8%
			Insulin	3(379)	0.20[0.05, 0.34]*	93.6%
			Biguanides + Sulfonylureas	3(266)	0.02[−0.17, 0.21]	81.0%
			Biguanides + a-Glucosidase	2(110)	−0.04[−0.12, 0.04]	21.1%
		Age of patients	41–64 years	17(1 986)	0.16[0.08, 0.25]*	94.6%
			>65 years	2(120)	0.01[-0.05,0.07]	0.0%
		FBG level at baseline	8–10 mmol/L	12(1,206)	0.13[0.04, 0.22]*	93.5%
			≥10 mmol/L	8(864)	0.12[0.02, 0.22]*	89.8%
		CM syndrome differentiation	*qi* and *yin* deficiency	7(637)	0.17[0.09, 0.25]*	75.5%
			*yin* deficiency and excessive heat	2(198)	0.16[−0.04, 0.35]	82.7%
		Disease duration	<5 years	6(762)	0.13[−0.04, 0.31]	94.3%
			≥5–10 years	5(542)	0.17[0.07, 0.27]*	84.9%
			≥10 years	5(559)	0.16[−0.00, 0.33]	95.7%
		BMI level at baseline	≥24–28 kg/m^2^	2(155)	0.03[−0.18, 0.23]	90.8%
	FINS (mU/Lor μIU/ml)	All studies	All studies	12(1,049)	−1.03 [−2.35, 0.29]	86.9%
		Risk of bias SG	Low risk of bias SG	5(413)	−1.60[−4.42, 1.21]	92.3%
		comparator Drug class	Biguanides	3(293)	−2.89[−6.96, 1.18]	95.7%
			Sulfonylureas	3(228)	−2.03[−3.21, −0.84]*	0.0%
			Biguanides + Sulfonylureas	1(150)	0.60[−1.16, 2.36]	NA
			Biguanides + a-Glucosidase	1(60)	−2.10[−2.79, −1.41]	NA
		Age of patients	41–64 years	10(929)	−1.46[−2.85, −0.07]*	88.2%
			>65 years	1(60)	1.32[-2.11,4.75]	NA
		FBG level at baseline	8–10 mmol/L	8(641)	-1.95[-3.45,-0.45]*	87.4%
			≥10 mmol/L	3(348)	1.62[0.09,3.15]	32.2%
		CM syndrome differentiation	*qi* and *yin* deficiency	3(215)	0.17[−2.08, 2.42]	47.4%
			*yin* deficiency and excessive heat	3(258)	−3.43[−7.37, 0.49]	95.3%
		treatment duration	≤3 months	11(911)	−1.41[−2.68, −0.15]*	84.5%
			≥6 months	1(138)	2.85[0.96, 4.73]	NA
		Disease duration	<5 years	5(378)	−2.96[−4.93, −0.99]	90.7%
			≥10 years	2(168)	−0.80[−4.34, 2.74]	70.1%
		BMI level at baseline	≥24–28 kg/m^2^	2(155)	−1.19[−3.21, 0.82]	81.8%
	IR	All studies	All studies	7(581)	−1.11[−1.44, −0.77]*	55.0%
		Risk of bias SG	Low risk of bias SG	4(353)	−1.14[−1.67, −0.60]*	72.6%
		comparator Drug class	Biguanides	3(293)	−0.80[−1.04, −0.56]*	0.0%
			Sulfonylureas	2(168)	−1.30[−1.85, −0.75]*	0.0%
			Biguanides + a-Glucosidase	1(60)	−0.98[−1.52, −0.44]*	NA
		FBG level at baseline	8–10 mmol/L	6(521)	−0.91[−1.12, −0.69]*	5.5%
		CM syndrome differentiation	*qi* and *yin* deficiency	1(95)	−0.90[−1.28, −0.52]	NA
			*yin* deficiency and excessive heat	3(258)	−0.79[−1.10, −0.48]*	3.5%
		Disease duration	<5 years	4(318)	−0.82[−1.08, −0.57]*	0.0%
			≥10 years	1(108)	−1.49[−2.23,-0.75]*	NA
		BMI level at baseline	≥24–28 kg/m^2^	2(155)	−0.93[−1.24, −0.62]*	0.0%
	IS	All studies	All studies	5(434)	0.09[-0.26,0.43]	95.0%
		comparator Drug class	Biguanides	2(210)	−0.14[−0.97, 0.69]	98.7%
			Sulfonylureas	2(168)	0.17[0.05, 0.28]*	0.0%
			Biguanides + Sulfonylureas	1(56)	0.56[0.05, 1.07]*	NA
		Age of patients	41–64 years	4(262)	0.23[0.12, 0.34]*	34.6%
		FBG level at baseline	8–10 mmol/L	3(224)	0.19[0.03, 0.36]*	35.4%
			≥10 mmol/L	2(210)	−0.14[−0.98, 0.69]	98.7%
		CM syndrome differentiation	*yin* deficiency and excessive heat	1(60)	0.21[0.06,0.36]*	NA
			Spleen deficiency	1(38)	0.28[0.18, 0.38]*	NA
		Disease duration	<5 years	3(154)	0.27[0.19, 0.35]*	0.0%
			≥5–10 years	1(172)	−0.57[−0.74, −0.40]	NA
			≥10 years	1(108)	0.09[−0.11, 0.29]	NA
		BMI level at baseline	≥24–28 kg/m^2^	1(38)	0.28[0.18,0.38]*	NA
	BMI (kg/m^2^)	All studies	All studies	4(233)	−0.45[−0.99, 0.08]	9.4%
		Risk of bias SG	Low risk of bias SG	2(135)	−0.38[−2.83, 2.07]	68.4%
CHM *vs.* lifestyle intervention	FBG (mmol/L)	All studies	All studies	1(72)	−0.62 [−1.28, 0.04]	NA
	2hPG (mmol/L)	All studies	All studies	1(72)	−2.14[−2.81, −1.47]*	NA
CHM as food *vs.* Lifestyle intervention	FBG (mmol/L)	All studies	All studies	1(90)	−1.08[−1.86, −0.30]*	NA
	2hPG (mmol/L)	All studies	All studies	1(90)	−1.23[−1.96, −0.50]*	NA
	TG (mmol/L)	All studies	All studies	1(90)	−0.30[−0.69, 0.09]	NA
	TC (mmol/L)	All studies	All studies	1(90)	−0.66[−1.10, −0.23]*	NA

*Statistically significant difference between groups.

CI, confidence interval; CM, Chinese medicine; FBG, fasting blood glucose; 2hPG, 2-hour postprandial blood glucose; HbA1c, hemoglobin A1c; TG, triglyceride; TC, total cholesterol; LDL, low density lipoprotein; HDL, high-density lipoprotein; FINS, fasting insulin; IR, insulin resistance; IS, insulin resistance index; BMI, body mass index; MD, mean difference; SG, sequence generation.

*2-h Postprandial Blood Glucose.* Forty-three RCTs including 4,004 participants assessed the effects of CHM plus hypoglycemic agents compared to hypoglycemic agents alone (27–33, 35, 36, 38–44, 46–52, 54–61, 64, 66–69, 71–77). All studies used the same hypoglycemic agent in both groups. Hypoglycemic agents included biguanides, sulfonylureas, *α*-glucosidase inhibitors, DPP-4 inhibitors, and insulins. Specific agents include metformin, gliclazide, glibenclamide, glimepiride, gliquidone, glipizide, pioglitazone, acarbose, voglibose, sitagliptin, and insulin. Treatment duration ranged from 4 to 24 weeks.

Meta-analysis results showed that as integrative medicine, CHM plus hypoglycemic agents was superior to hypoglycemic agents alone at reducing 2hPG levels at the end of treatment [MD −1.46 (−1.73, −1.20), I^2^ = 86.2%]. Heterogeneity remained high after sensitivity analysis with studies with low risk of bias for sequence generation [nine studies, n = 895, MD −1.64 (−2.27, −1.02); I^2^ = 91.8%] (31, 32, 38, 43, 44, 46, 64, 69, 73) (**Table 2**).

Grouping of studies using metformin produced the biggest pool with 16 RCTs and 1,572 participants. The effect on 2hPG is similar to the overall result with reduced heterogeneity [MD −1.46 (−1.73, −1.20); I^2^ = 44.3%] (28, 39, 40, 43, 47, 52, 54, 57, 59–61, 64, 68, 73, 74, 77). Combinations of different drug classes also showed a significant difference between groups, but heterogeneity remained high. Additional subgroup analyses based on patient age groups, baseline levels of FBG, CM syndrome differentiation, treatment duration, disease duration, and baseline levels of BMI all showed significant differences between groups (**Table 2**).

*Hemoglobin A1c*. Thirty-five RCTs including 3,009 participants assessed the effects of CHM plus hypoglycemic agents *versus* hypoglycemic agents alone (27–32, 34, 37–45, 47–49, 51–55, 57, 59, 60, 64, 67, 68, 70, 72–77). All studies used the same hypoglycemic agents in both groups, including biguanides, sulfonylureas, *α*-glucosidase inhibitors, and insulins. Specific agents include metformin, gliclazide, glipizide, gliquidone, voglibose, acarbose, and insulin. Treatment duration ranged from 2 to 24 weeks.

CHM plus hypoglycemic agents was superior to hypoglycemic agents alone [MD −0.84 (−1.05, −0.64), I^2^ = 88.2%]. Heterogeneity remained high in subgroup analysis except in two subgroups where sulfonylureas or biguanides plus sulfonylureas were combined with CHM (**Table 2**). In two RCTs with 120 participants, the result indicated that the value of HbA_1c_ was reduced in people receiving CHM plus sulfonylureas compared to sulfonylureas alone [MD −0.65 (−1.07, −0.24), I^2^ = 0.0%] (49, 55). CHM plus biguanides and sulfonylureas were used as comparator in three RCTs, including 196 participants; the result showed that in the integrative medicine group, HbA_1c_ significantly lower compared to pharmacotherapy group alone [MD −0.35 (−0.68, −0.02), I^2^ = 0.0%] (27, 29, 75).

###### Blood Lipid Metabolism Indicators

*Total Cholesterol.* Twenty-four RCTs including 2,582 participants assessed the effects of CHM plus hypoglycemic agents *versus* hypoglycemic agents alone (27–31, 34, 35, 37, 39, 42–45, 54, 56, 58, 63, 64, 66, 70–73, 77). All studies used the same hypoglycemic agents in both groups; specific agents include metformin, glibenclamide, glipizide, glimepiride, acarbose, and insulin. Treatment duration ranged from 2 to 12 weeks.

Meta-analyses showed that CHM in addition to hypoglycemic agents was superior to hypoglycemic agents alone at reducing TG levels in T2DM patients [MD −0.40 (−0.51, −0.29), I^2^ = 79.2%]. Meta-analysis of studies assessed as low risk for sequence generation produced a similar result that was more homogeneous [seven RCTs, 792 participants, MD −0.36 (−0.50, −0.22); I^2^ = 50.5%] (31, 34, 37, 43, 44, 64, 73).

Subgroup analyses by patient age groups, FBG level at baseline, disease duration and BMI levels at baseline showed similar effects on TG (**Table 2**). Combined use of CHM with biguanides, insulin, biguanides plus sulfonylureas and biguanides plus a-Glucosidase also produced significant difference between groups; results are like the overall analysis (**Table 2**).The combination of CHM to a sulfonylureas did not produce a better result than these agents alone (56, 71) (**Table 2**). In studies that provided details on CM syndrome differentiation, adding CHM to hypoglycemic agents showed more benefit in reducing TG levels in patients with *qi* and *yin* deficiency (29, 31, 34, 35, 37, 39, 71, 77), *yin* deficiency with excessive heat (43, 64), but not those with spleen deficiency (28) (**Table 2**).

*Triglyceride.* Twenty-four RCTs including 2,582 participants assessed the effects of CHM plus hypoglycemic agents *versus* hypoglycemic agents alone (27–31, 34, 35, 37, 39, 42–45, 54, 56, 58, 63, 64, 66, 70–73, 77). All studies used the same hypoglycemic agents in both groups; specific agents include metformin, glibenclamide, glipizide, glimepiride, acarbose, and insulin. Treatment duration ranged from 2 to 12 weeks.

Meta-analyses showed that CHM in addition to hypoglycemic agents was superior to hypoglycemic agents alone at reducing TC levels in T2DM patients [MD −0.40 (−0.58, −0.22), I^2^ = 95.2%]. Meta-analysis of studies assessed as low risk of bias for sequence generation showed no difference between groups [seven RCTs, 792 participants, MD −0.34 (−0.86, 0.19); I^2^ = 98.3%] (31, 34, 37, 43, 44, 64, 73).

Subgroup analysis showed that the combination of CHM with sulfonylureas (56, 71), insulin (31, 35, 44), or biguanides plus sulfonylureas (27, 29, 58) was not superior at reducing TC levels (**Table 2**). Studies with patient’s age group of 41–61years (20 RCTs, n = 2,230) (27–31, 34, 35, 42–44, 56, 58, 63, 64, 66, 70–73, 77) showed a significant difference between the two groups [MD −0.39 (−0.59, −0.18); I^2^ = 96.0%] but not in the two studies with patients who are older [n = 120, MD −0.46 (−0.99, 0.08); I^2^ = 57.8%] (37, 45). Subgroup analyses by FBG level at baseline showed similar effects on TC. Analysis on studies that provided CM syndrome information showed that integrative medicine was superior to hypoglycemic agents alone in patients with *qi* and *yin* deficiency (29, 31, 34, 35, 37, 39, 71, 77), but not those with *yin* deficiency and excessive heat (43, 64) or spleen deficiency (28) (**Table 2**). Studies with disease duration was 5–10 years [six RCTs, n = 612, MD −0.63 (−0.77, −0.50); I^2^ = 76.8%] (29, 54, 66, 70, 71) showed a significant difference between the two groups but not in the study with disease duration of less than 5 years (27, 28, 43, 56, 63, 64, 72) or equal or more than 10 years (31, 37, 44, 45, 73, 77) disease duration subgroup (**Table 2**). Additional subgroup analyses by BMI level at baseline showed no difference between groups (**Table 2**).

*Low Density Lipoprotein and High-Density Lipoprotein.* Twenty-one RCTs including 2,338 participants assessed the effects of CHM plus hypoglycemic agents *versus* hypoglycemic agents alone (27, 29–31, 34, 35, 37, 39, 43–45, 54, 56, 58, 63, 64, 66, 70–73). Different classes of hypoglycemic agents were used, and treatment duration ranged from 2 to 12weeks.

Meta-analysis of 21 studies showed that CHM plus hypoglycemic agents was superior to hypoglycemic agents alone at reducing LDL levels at the end of treatment [MD −0.45 (−0.65, −0.27); I^2^ = 97.4%]; however, heterogeneity was high. Seven studies were assessed as low risk of bias for sequence generation, subgroup analysis showed similar results to the overall studies (31, 34, 37, 43, 44, 64, 73). Subgroup analysis by drug class showed that CHM added to sulfonylureas (56, 71) or insulin (31, 35, 44) was no superior at reducing LDL levels compared to sulfonylureas or insulin alone (**Table 2**). Subgroup analyses by FBG level at baseline, disease duration or BMI level at baseline showed similar effects on LDL. Studies with age group of 41–64 years showed significant between group results (27, 29–31, 34, 35, 43, 44, 56, 58, 63, 64, 66, 70–73). In studies that provided details on CM syndrome differentiation, subgroup analysis showed that CHM is better at reducing LDL levels in patients with *qi* and *yin* deficiency (29, 31, 34, 35, 37, 39, 71), but not in those with *yin* deficiency and excessive heat (43, 64) (**Table 2**).

Meta-analysis result of 21 studies showed that CHM plus hypoglycemic agents was superior to hypoglycemic agents alone at improving HDL levels [MD 0.15 (0.08, 0.22); I^2^ = 93.9%]; however, heterogeneity was high. Results from studies with a low risk of bias for sequence generation produced a similar result (31, 34, 37, 43, 44, 64, 73) (**Table 2**). Subgroup analysis by drug class showed that CHM in combination with biguanides (34, 39, 43, 54, 63, 64, 70, 73), or insulin (31, 35, 44) can improve HDL levels better than using the agents alone (**Table 2**). Studies with patient age group of 41–64 years showed significant between group results (27, 29–31, 34, 35, 43, 44, 56, 58, 63, 64, 66, 70–73) but not in two studies with older age groups (37, 47). Subgroup analysis using FBG level at baseline showed significant differences between groups with high heterogeneity (**Table 2**). In T2DM patients with *qi* and *yin* deficiency (29, 31, 34, 35, 37, 39, 71), integrative medicine was better at improving HDL levels than hypoglycemic agents alone (**Table 2**). Studies with disease duration was 5–10 years (five RCTs, n = 542) (29, 54, 66, 70, 71) showed a significant difference between the two groups but not in studies with a disease duration of less than 5 years (27, 43, 56, 63, 64, 72) or more than 10 years (31, 37, 44, 45, 73) (**Table 2**). Additional subgroup analyses by BMI level at baseline showed no difference between groups (**Table 2**).

###### β-Cell Function Indicators

*Fasting Insulin.* Twelve RCTs including 1,049 participants assessed the effects of CHM plus hypoglycemic agents *versus* hypoglycemic agents alone (33, 34, 43, 45, 51, 55, 58, 64, 65, 69, 72, 76). Different classes of hypoglycemic agents were included and treatment duration ranged from 4 to 24 weeks.

At the end of treatment, CHM plus hypoglycemic agents was not superior to hypoglycemic agents alone at reducing FINS levels [MD −1.03 (−2.34, 0.29), I^2^ = 86.9%]. Meta-analysis of results from studies with a low risk of bias for sequence generation showed similar results from the overall result (34, 43, 64, 65, 69). Subgroup analysis by hypoglycemic agent class showed varied results for different class of drugs; there were benefits seen in the addition of CHM to sulfonylureas but not in other hypoglycaemic agents (**Table 2**). Studies with patient age group of 41–64 years showed significant difference between group results (33, 34, 43, 51, 55, 58, 64, 65, 72, 76) but not in older subgroup (45). Subgroup analysis based on FBG level at baseline, the lower reading of 8–10 mmol/L subgroup (33, 34, 43, 55, 64, 69, 72, 76) showed more benefit but not in higher subgroup (45, 51, 58). Studies with a shorter treatment duration of less than 3 months showed benefit in adding CHM to hypoglycaemic agents (33, 34, 43, 45, 55, 58, 64, 65, 69, 72, 76), but not in studies with a longer treatment (51). Additional subgroup analyses by CM syndrome differentiation, disease duration and BMI level at baseline showed no difference between groups (**Table 2**).

*Homeostatic model assessment of insulin resistance.* Seven RCTs including 581 participants assessed the effects of CHM plus hypoglycemic agents *versus* hypoglycemic agents alone (33, 34, 43, 64, 65, 72, 76). Hypoglycemic agents included biguanides, sulfonylureas, *α*-glucosidase inhibitors, and insulins. Treatment duration ranged from 4 to 12 weeks.

CHM plus hypoglycemic agents was superior to hypoglycaemic agents alone [MD −1.11 (−1.44, −0.77); I^2^ = 55.0%]. Meta-analysis of results from studies with a low risk of bias for sequence generation showed similar results (34, 43, 64, 65); however, heterogeneity was high. Subgroup analyses based on drug class, baseline levels of FBG, disease duration and baseline levels of BMI all showed significant differences between groups (**Table 2**). Subgroup analysis by CM differentiation showed benefit of adding CHM to hypoglycemic group than hypoglycemic agents alone in patients with *yin* deficiency and excessive heat (33, 43, 64) (**Table 2**).

*Insulin resistance index.* Five RCTs including 434 participants assessed the effects of CHM plus hypoglycemic agents *versus* hypoglycemic agents alone (27, 28, 33, 54, 76). Various hypoglycemic agents were used including metformin, gliclazide, and glimepiride. All studies had a treatment duration of less than or equal to 12 weeks; the shortest treatment duration was 4 weeks.

Meta-analysis showed that CHM plus hypoglycemic agents was not superior to hypoglycemic agents alone [MD 0.09 (−0.26, 0.43), I^2^ = 95.0%]. Subgroup analysis by drug class showed benefit in adding CHM to sulfonylureas (33, 76) and the combination of biguanides with sulfonylureas (27). No significant difference was observed when CHM was added to biguanides (28, 54). Subgroup analysis using age of patients of 41–64 years showed benefit (27, 28, 33, 76). Subgroup analysis based on lower FBG level at baseline (8–10 mmol/L) (27, 33, 76) showed more benefit but not in the higher subgroup (28, 54). In studies that provided information on CM differentiation, meta-analyses showed a benefit in adding CHM to hypoglycemic agents in patients with *yin* deficiency and excessive heat (33) or spleen deficiency (28). Studies with disease duration of less than 5 years (three RCTs, n = 154) (27, 28, 33) showed a significant difference between the two groups but not those studies with more than 5 years of disease duration (54, 76). Additional subgroup analyses by BMI level at baseline showed difference between groups (28) (**Table 2**).

*Body Mass Index.* Four RCTs including 233 participants assessed the effects of CHM plus hypoglycemic agents ***versus*** hypoglycemic agents alone (28, 34, 62, 72). Hypoglycemic agents included biguanides, ***α***-Glucosidase inhibitors and insulins. Specific agents include metformin, acarbose, and insulin. Treatment duration ranged from 8 to 12 weeks.

Meta-analysis results showed that CHM plus hypoglycemic agents was not superior to hypoglycemic agents alone at improving BMI [MD −0.45 (−0.99, 0.08); I^2^ = 9.4%). Meta-analysis of studies assessed as low risk for sequence generation produced a similar result [two studies, 135 participants, MD −0.38 (−2.83, 2.07); I^2^ = 68.4%] (34, 62), and no difference was found between groups.

##### CHM Plus Lifestyle Intervention Versus Lifestyle Intervention Alone

One RCT with 72 participants compared CHM (including *Shanyao*) plus lifestyle intervention with lifestyle intervention for 4 weeks (26). The result showed CHM together with lifestyle intervention was superior to lifestyle intervention alone in reducing 2hPG and TC levels at the end of treatment [MD −2.14 (−2.81, −1.47), −0.66 (−1.10, −0.23)]; there was no difference in the FBG and TG levels between the two groups [MD −0.62 (−1.28, 0.04), −0.30 (−0.69, 0.09)].

##### CHM Diet Therapy Plus Lifestyle Intervention Versus Lifestyle Intervention Alone

One RCT (n = 90) compared CHM as diet therapy (including *Shanyao*) with lifestyle intervention with a treatment duration of 12 weeks (25). The result showed that combination of Chinese diet therapy using *Shanyao* and lifestyle intervention was superior to lifestyle intervention alone at reducing FBG and 2hPG levels at the end of treatment [MD −1.08 (−1.86, −0.30), −1.23 (−1.96, −0.50)].

##### Adverse Events

Out of the 53 studies, 21 studies reported on AEs. Of these, 10 studies provided specific details about the AEs.

*CHM Plus Hypoglycaemic Agents vs. Hypoglycaemic Agents.* In twenty RCTs of CHM plus hypoglycemic drugs *versus* hypoglycemic drugs, ten studies reported no AEs (28, 30, 32, 41, 51, 52, 55, 72, 76, 77), ten studies provided specific details about AEs (29, 37, 43–46, 53, 56, 58, 64). In the integrative medicine group, the most common AEs were hypoglycemia (four cases), nausea (three cases), hypertension (two cases), insomnia (one case), epigastric discomfort (two cases), diarrhea (two cases), frequency of urine (three cases), drug-related adverse reactions (one case unknown). There were three cases of diarrhea reported; however, it was not clear whether the AEs were from the treatment group or the control group (43).

Twenty-four AEs were reported in the hypoglycemic drug group. In the hypoglycemic agents group, hypoglycemia (seven cases) was the most common AE. Other AEs included nausea (three cases), headache (two cases), stomach distention (three cases), dyspepsia (two cases), fatigue (two cases), rash (one case), and four other AEs were not described in detail.

One study that compared CHM plus lifestyle intervention to lifestyle intervention reported no AEs (26).

##### Assessment Using GRADE

An assessment of the quality of the evidence from RCTs was undertaken using GRADE. Interventions, comparators, and outcomes included were selected based on a consensus process. Comparisons were: CHM plus hypoglycemic agents *versus* hypoglycemic agents, CHM plus lifestyle intervention *versus* lifestyle intervention and CHM diet therapy plus lifestyle intervention *versus* lifestyle intervention.

Evidence of *Shanyao* formulae for T2DM was low to moderate quality (**Table 3**). The results showed that oral formulae containing *Shanyao* may improve glycolipid metabolism and fasting insulin level.

**Table 3:** GRADE: Quality of the evidence of *Shanyao* formulae for T2DM.

Outcomes	№ of participants(studies)Follow-up	Certainty of the evidence(GRADE)	Anticipated absolute effects	
***CHM plus Hypoglycemic agents vs. Hypoglycemic agents***			**Risk with [Hypoglycemic drugs]**	**Risk difference with [CHM plus hypoglycemic drugs]**
Fasting blood glucose (FBG) Treatment duration: mean 8.85 weeks	4,375 (48 RCTs)	⨁⨁⨁◯ MODERATE^a^	The mean fasting blood glucose was **7** **.43** mmol/L	MD **0.93 mmol/L lower** (1.1 lower to 0.76 lower)
2-hour Postprandial blood glucose (2hPG) Treatment duration: mean 8.79 weeks	4,004 (43 RCTs)	⨁⨁⨁◯ MODERATE^a^	The mean postprandial blood glucose was **10.44** mmol/L	MD **1.46 mmol/L lower** (1.73 lower to 1.2 lower)
Glycosylated Hemoglobin A1c (HbA1c) Treatment duration: mean 9.32 weeks	3,009 (35 RCTs)	⨁⨁◯◯ LOW ^a,b^	The mean glycosylated Hemoglobin A1c was **7.44%**	MD **0.84% lower** (1.05 lower to 0.64 lower)
Triglyceride (TG) Treatment duration: mean 9.04 weeks	2,582 (24 RCTs)	⨁⨁◯◯ LOW ^a,b^	The mean triglyceride was **2.41** mmol/L	MD **0.4 mmol/L lower** (0.51 lower to 0.29 lower)
Cholesterol (TC) Treatment duration: mean 9.04 weeks	2,582 (24 RCTs)	⨁⨁⨁◯ MODERATE^a^	The mean cholesterol was **5.15** mmol/L	MD **0.4 mmol/L lower** (0.58 lower to 0.22 lower)
Fasting insulin (FINS) Follow-up: range 1 to 30 weeks Treatment duration: mean 10.67 weeks	1,049 (12 RCTs)	⨁⨁◯◯ LOW ^a,b^	The mean fasting insulin was **15.11** μU/ml	MD **1.03 μU/ml lower** (2.35 lower to 0.29 higher)
***CHM plus lifestyle intervention vs. lifestyle intervention***			**Risk with [lifestyle intervention]**	**Risk difference with [CHM]**
Fasting blood glucose (changed from baseline)(FBG) Treatment duration 4 weeks	72 (one RCT)	⨁⨁⨁◯ MODERATE^c^	The mean fasting blood glucose (changed from baseline) was −**0.32** mmol/L	MD **0.62 mmol/L lower** (1.28 lower to 0.04 higher)
2-hour Postprandial blood glucose(changed from baseline)(2hPG) Treatment duration 4 weeks	72 (one RCT)	⨁⨁⨁◯ MODERATE^c^	The mean postprandial Blood Glucose(changed from baseline) was −**0.77** mmol/L	MD **2.14 mmol/L lower** (2.81 lower to 1.47 lower)
***CHM diet therapy plus lifestyle intervention vs. lifestyle intervention***			**Risk with [lifestyle intervention]**	**Risk difference with [CHM]**
Fasting blood glucose (changed from baseline)(FBG) Treatment duration 12 weeks	90 (one RCT)	⨁⨁⨁◯ MODERATE^c^	The mean fasting blood glucose (changed from baseline) was −**0.87** mmol/L	MD **1.08 mmol/L lower** (1.86 lower to 0.30 lower)
2-hour Postprandial blood glucose(changed from baseline)(2hPG) Treatment duration 12 weeks	90 (one RCT)	⨁⨁⨁◯ MODERATE^c^	The mean postprandial Blood Glucose(changed from baseline) was **−1.34** mmol/L	MD **1.23 mmol/L lower** (1.96 lower to 0.50 lower)
Triglyceride(changed from baseline)(TG) Treatment duration 12 weeks	90 (one RCT)	⨁⨁⨁◯ MODERATE^c^	The mean triglyceride(changed from baseline) was **−0.41** mmol/L	MD **0.3 mmol/L lower** (0.69 lower to 0.09 higher)
Cholesterol(changed from baseline)(TC) Treatment duration 12 weeks	90 (one RCT)	⨁⨁⨁◯ MODERATE^c^	The mean cholesterol(changed from baseline) was **−0.45** mmol/L	MD **0.66 mmol/L lower** (1.1 lower to 0.23 lower)

*The risk in the intervention group (and its 95% confidence interval) is based on the assumed risk in the comparison group and the relative effect of the intervention (and its 95% CI).

CHM, Chinese herbal medicine; CI, Confidence interval; GRADE, Grading of Recommendations Assessment, Development and Evaluation; MD, Mean difference; RCTs, randomized controlled trials;

Explanations

^a^High statistical heterogeneity, p < 0.05; ^b^Funnel plot not symmetrical; ^c^Small sample size. Bold values is used to highlight.

### Experiment Research Evidence of *Shanyao* for Diabetes

The major component groups of *Shanyao* are saponins, phenolic compounds, sterols, and mucilage (8). Identified active ingredients of *Shanyao* include polysaccharides, flavonoids, allantoin, choline, dioscin, and so on (9–14). *Shanyao* has shown immunomodulatory and anti-inflammatory effects (78, 79). Hpyoglycemic effects of *Shanyao* as food and pharmacological effects in relation to T2DM are reviewed below.

#### Nutritional Study on the Hypoglycemic Effect of *Shanyao*

*Shanyao* has been studied to explore its effect on blood glucose as a food. A study indicated that meal A (maize flour meal) was composed of 81% carbohydrate, 3% protein, and 11% fat; meal B (cassava flour meal) was composed of 76% carbohydrate, 3% protein, and 15% fat; while meal C (yam flour meal) was composed of 85% carbohydrate, 2% protein, and 8% fat. Analysis of the results demonstrated a better glycemic response with meals A and C compared with meal B; *Shanyao* as food may bring more benefits to blood glucose (80).

*Shanyao*’s glycemic index has also been investigated. Glycemic index was introduced to rank how slowly or quickly carbohydrate containing foods are digested and increase postprandial blood glucose (24). A study has shown that the actual calorie input may be much lower in *Shanyao* than in brown rice and white bread, showing that the glycemic value of *Shanyao* is lower than brown rice (81), which is beneficial for controlling postprandial blood glucose, in turn may benefit diabetic patients.

An experimental research conducted in Brazil showed that *Shanyao* flour alleviated the consequences of the experimental diabetic disease. It showed that *Shanyao* flour could control the rise in blood glucose levels in diabetic rats and significantly greater radiodensity of femoral head when compared to DM group, suggesting protection in oxidative agents and postpone bone damage caused by diabetes (82).

Different *Shanyao* species are present depending on place of production. *Dioscorea alata* is known to have the highest yields among the *Shanyao* species with tubers. *Dioscorea alata* has shown to have higher amylose and total dietary fiber contents, resulting in slower absorption rates and can be particularly useful in diets for diabetics (83).

#### Pharmacological Effects in Relation to Diabetes of *Shanyao* and Its Compounds

The pharmacological effects and preventative effects for T2DM of *Shanyao* or its compounds have been tested in various animal models.

As we mentioned earlier on, a popular formula used for DM is Liu wei di huang wan made up of six herbs including *Shanyao*. In fructose-rich chow fed rats, after feeding of Liu wei di huang wan at 26 mg/kg for 60 min, reduction in plasma glucose was observed (84). When *Shanyao* was removed from the mixture, plasma glucose was not modified while this action was not modified by the removal of the other five herbs, indicating important hypoglycemic roles of *Shanyao* (84). Further, *Shanyao* produced similar hypoglycemic effects of the Liu wei di huang wan formula, while other herbs in the formula failed to produce the same effects. The authors also investigated the role of *Shanyao* in improving insulin sensitivity and found that oral administration of *Shanyao* at 4.2 mg/kg three times daily into streptozotocin-induced diabetic rats increased the response to exogenous insulin (84).

In vehicle-controlled mice and in alloxan-induced diabetic mice, *Shanyao* decoction concentrate at 300 mg and 600 mg/kg for 10 consecutive days can significantly reduce blood glucose level (85). Further, *Shanyao* has shown preventative effects on induced blood glucose elevation due to different causes including adrenaline, alloxan, and glucose feeding (85). As for hypoglycemic effects, *Shan yao* can reduce total cholesterol and triglyceride levels in diabetic rats, showing lipid lowering effects (86).

##### Polysaccharides

In alloxan-induced diabetic rats and mice, high dose DOTP-80 water-soluble polysaccharide (400 mg/kg) had strong hypoglycemic activity (87). Moreover, water-soluble polysaccharide could increase the level of antioxidant enzyme (superoxide dismutase) activity in alloxan-induced diabetic mice and stimulated an increase in glucose disposal in diabetic rats (87).

In high fat fed streptozotocin-induced type 2 diabetic rats, *Shanyao* polysaccharide administration significantly reduced fasting plasma glucose levels, increased serum insulin levels, and decreased glucagon levels, showing hypoglycemic effects (88, 89). The hypoglycemic effects of *Shanyao* polysaccharides are comparable to metformin (88, 89).

*In vitro* testing of *Shanyao* polysaccharide (YP-1) from a variety of Chinese yam revealed that YP-1 could stimulate ConA-induced T lymphocyte proliferation, and its branches are extremely important for the expression of the enhancement of the immunological activity (90). Considering the important role of the immune system in the progression of T2DM, YP-1 could play a part in enhancing immunological activities in T2DM.

*Shanyao* polysaccharides have shown antioxidant activities. Purified *Shanyao* polysaccharide could scavenge hydroxyl radical and superoxide radical. Additionally, it displayed inhibitory activity against *E. coli*, with a minimal inhibitory concentration of 2.5 mg/ml (91).

Dexamethasone-induced insulin resistance glucose/lipid metabolism diabetic mice model was established to evaluate the hypoglycemic effect of different concentrations of *HuaiShanyao* and different molecular weights of polysaccharide HSY-I, HSY-II, and HSY-III. The results indicated that the Chinese yam polysaccharide mixture had hypoglycemic effect (92).

##### Saponins and Flavones

The metabolic syndrome is a term for cluster of multiple metabolic risk criteria which is positively correlated with type 2 diabetes mellitus. *Shanyao* dioscorin interventions exhibit improved metabolic syndrome activities in obese rats, and peptic hydrolysates of *Shanyao* dioscorin *in vitro* exhibit DPP IV inhibitory activities (93).

After intragastric administration of dioscoreae flavone in diabetic mice, it was found that blood glucose of diabetic mice was decreased, the amount of drinking water decreased, and the weight of mice recovered (94). Further experiments showed that dioscoreae flavone could inhibit *α*-glucosidase activity, effectively reduce superoxide dismutase activity and malondialdehyde content in diabetic mice, and have antioxidant effect (94).

Experimental results show that the saponins and flavones of *Shanyao* had a marked inhibition effect on *α*-amylase. The kinetic analysis indicates that the inhibition type of saponins and flavones on *α*-amylase was competitive, and the enzyme-substrate apparent dissociation constant (Km′) of acarbose (the control), saponins, and flavones is 118.86, 79.23, 49.51 mg/ml, respectively (95).

##### Allantoin

Allantoin is known as the active principle richly contained in *Shanyao* (Dioscorea spp.). It is identified as an abundant and active component in *Shanyao* (Dioscorea spp.) (96). Allantoin may improve glucose utilization in the skeletal muscle through *β*-endorphin dependent- and independent-pathways that decrease plasma glucose in STZ-diabetic rats (97).

Allantoin can increase *β*-endorphin release through activation of I-2A receptors to lower blood glucose. Also, allantoin can activate I-2B receptors in the skeletal muscle or adipose tissues and brain and others to reduce blood glucose in STZ-diabetic rats. Both actions of allantoin may assist the increase of insulin sensitivity in diabetic rats (98).

##### Ligands

*α*-Glucosidase inhibitors are widely used in the treatment of patients with T2DM, which delay the absorption of carbohydrates from the small intestine and result in lowered postprandial blood glucose and insulin levels (99). Two ligands identified as 2,4-dimethoxy-6,7-dihydroxyphenanthrene and batatasin I were extracted from *Shanyao* using *α*-glucosidase functionalized magnetic nanoparticles as a solid phase extraction absorbent. Their *α*-glucosidase inhibitory activities were significantly higher than acarbose (100).

## Discussion

### Summary of Evidence

In this study, we searched clinical studies and experimental studies to present an overall picture of the evidence for *Shanyao* and its formulae for additional benefits to conventional therapies in the management of DM. We also explored the possible mechanisms of actions for *Shanyao*.

This study presents a comprehensive and up-to-date evidence for the treatment of T2DM with *Shanyao* formulae. A systematic review of 53 randomized controlled trials was identified in English- and Chinese-language databases, and meta-analysis was conducted to evaluate the additive benefits and safety of *Shanyao* formulae. Systematic evaluation of modern literatures suggested that in terms of controlling blood glucose, blood lipid and improving insulin resistance, traditional Chinese medicine formulae containing *Shanyao* combined with conventional therapies do show added benefits when compared to hypoglycemic agents and lifestyle intervention alone.

Systematic review of clinical studies indicates that *Shanyao*-containing herbal formulae can improve important outcome measures for T2DM including FBG, 2hPG, HbA_1c_, TG, TC, and IR in people with T2DM. Further, the adverse events in the herbal formula group are lower than in the control group, suggesting a good safety profile. Taken together, Chinese herbal medicine therapy including *Shanyao* can be of beneficial for patients with T2DM. The heterogeneity in our meta-analysis was high. We performed a subgroup analysis based on pre-set conditions, but the results of subgroup analysis did not fully explain the source of heterogeneity. Considering different herb usages in different clinical trials, this could be a possible source of heterogeneity in the meta-analysis. Therefore, it is necessary to interpret these results with caution.

The occurrence of DM in the current day and age is closely related to lifestyle change. We found that *Shanyao* 山药, *Fuling* 茯苓, *Maimendong*麦门冬, *Shanzhuyu* 山茱萸, *Haungqi* 黄芪, *Shengdihuang* 生地黄, *Gegen* 葛根, *Danshen* 丹参, *Tianhuafen* 天花粉, and *Huanglian* 黄连are used more in modern literatures. The use of Chinese herbs that taste sweet and bitter, has the effect of invigorating *qi* and nourishing *yin*, infiltrating dampness and solidifying astringency, clearing heat, generating body fluid, and detoxifying. In recent years, the prevalence of diabetes in obese and overweight people has doubled in modern China (6). TCM believes that phlegm-dampness is the basic cause of obesity. An ancient practitioner Zhu Danxi clearly put forward in “*Dan Xi Zhi Fa Xin Yao*丹溪治法心要” that “people who are obese have excessive dampness and phlegm in the body”. Obesity combined with diabetes is characterized by *yin* deficiency, internal heat, and phlegm dampness. Therefore, modern diabetes treatment focuses on nourishing *yin* and clearing heat, drying dampness and resolving phlegm.

To sum up the experimental evidence, *Shanyao* as a food, is rich in dietary fiber with low glycemic index, which is beneficial to improving postprandial blood glucose of diabetic patients. In diabetic animals, *Shanyao* can reduce blood sugar, regulate blood lipid, resist oxidation and be beneficial to the bone. The main active ingredients of *Shanyao* can regulate blood glucose by improving insulin resistance, inhibiting *α*-glucosidase activity, delaying the absorption of glucose in intestine, inhibiting DPP-IV activity, increasing the concentration of endogenous GLP-1, antioxidation and regulating immunity.

### Limitations of the Current Review

The included clinical studies also present methodological shortfalls. In randomized clinical trials, appropriate randomization and allocation concealment methods can reduce bias, but only 25.81% of the included studies described the randomization methods, and only 2.58% of the studies described allocation concealment. Inappropriate randomization and allocation concealment may exaggerate the efficacy. Inadequate blinding method can also lead to overestimation of the effect. Chinese herbal medicine has many obstacles to the implementation of blinding in clinical trials due to the herbal preparation and odor; it is easy for participants to identify which group they are in. Active exploration of the preparation of placebo and the implementation of blinding method of traditional Chinese medicine may be a way to solve this problem. In addition, the included studies did not publish study protocols, and the non-standardization of clinical research reports is not helpful to the dissemination and recognition of research results.

Modern literature systematic reviews included the study of traditional Chinese medicine formulae containing *Shanyao*. The included studies all used *Shanyao* as part of a traditional Chinese medicine formula for T2DM. Therefore, the results of modern literature analysis do not fully represent the role of *Shanyao* in the treatment of T2DM in reducing blood sugar, lipid and improving IR.

### Implications in Research and Clinical Practice

Future clinical studies should be recommended to design and report data following the items required by the Consolidated Standards of Reporting Trials (CONSORT) (101) and its extensions for herbal medicine and traditional CM (102, 103).

Rigorous methodology is recommended when designing future clinical trials with correct methods of sequence generation and allocation concealment. Protocols should be published and be registered to minimize reporting bias and increase transparency in the reporting the results.

The included studies reported outcome measures directly related to blood glucose metabolism, lipid profiles, and *β*-cell function. As we know, T2DM is a chronic and progressive disease, and those who are affected may have drastic lifestyle changes. Other clinically important outcomes such as quality of life would provide another aspect of understanding the effect of CM therapies for T2DM.

As we all know, T2DM is a progressive and lifelong disease. The included clinical studies had treatments between 2 and 24 weeks with only few studies reporting on follow-up data. Future studies may consider using longer treatment durations and lengthy follow-up period to reflect clinical practice and provide evidence for the long-term effects of CM treatments using *Shanyao*.

Although we present in the study available experimental evidence for *Shanyao*, the volume of studies is small compared to other commonly used herbs such as ginseng. More experimental study data will improve the understanding of mechanisms of action of hypoglycemic effects of *Shanyao*. Results may provide future directions for the development of new and improved hypoglycemic agents.

Based on clinical studies, herbs that are commonly used with *Shanyao* in the modern context include *huangqi* 黄芪, *shengdihuang* 生地黄, *gegen* 葛根*, tianhuafen* 天花粉, *danshen* 丹参*, fuling* 茯苓, *maimendong* 麦门冬*, shanzhuyu 山茱萸*, and *huanglian* 黄连.

Finally, this study provides comprehensive information about *Shanyao*’s use for T2DM in clinical studies. At the same time, it explores possible mechanisms through experimental studies, providing evidence of hypoglycemic mechanism of *Shanyao* at a cellular and animal model levels.

## Data Availability Statement

The raw data supporting the conclusions of this article will be made available by the authors, without undue reservation.

## Author Contributions

All authors listed have made a substantial, direct, and intellectual contribution to the work and approved it for publication.

## Funding

The China–Australia International Research Centre for Chinese Medicine (CAIRCCM)—a joint initiative of RMIT University, Australia and the Guangdong Provincial Academy of Chinese Medical Sciences, China supported this research. We also received funding from the Ministry of Science & Technology of China (grant number 2015BAI04B00) and Guangdong Provincial Hospital of Traditional Chinese Medicine (grant numbers 2018DB03).

## Conflict of Interest

The authors declare that the research was conducted in the absence of any commercial or financial relationships that could be construed as a potential conflict of interest.
